# Relationship between DMFT index and reproductive history- a cross-sectional study on enrollment phase of Azar cohort study

**DOI:** 10.1186/s12903-022-02578-4

**Published:** 2022-11-19

**Authors:** Sahra Hefzollesan, Nasrin Sharififard, Zeinab Mahboobi, Elnaz Faramarzi

**Affiliations:** 1 Liver and Gastrointestinal Diseases Research Center, Tabriz University of Medical Sciences, Tabriz, Iran; 2 Department of Community Oral Health, School of Dentistry, Tabriz University of Medical Sciences, Golgasht st, Tabriz, 5166614711 Iran

**Keywords:** Pregnancy, Azar cohort, Menstruation, Menopause, DMFT

## Abstract

**Background:**

Hormonal changes in women throughout life might affect the oral health. The aim of this study is to investigate the relationship between the Decayed, Missing, and Filled Teeth (DMFT) index and reproductive history.

**Methods:**

The present cross-sectional study was performed using data of Azar Cohort Study conducted in 2014, in Shabestar city, East Azerbaijan Province, Iran. In the present study, the data of all 8294 women from the enrollment phase of the Azar cohort were included. All available data related on the variables of reproductive history (including age at the onset of menstruation, age of onset of menopause, age of first pregnancy, and frequency of pregnancy), age at interview, educational level, socioeconomic status, frequency of tooth brushing, chronic diseases, body mass index and DMFT were extracted. Negative binomial regression with loglink was used to analyze the relationship between variables. Three regression models have been applied to adjust the effect of confounding variables. Model 1 adjusted for education, socio-economic status, age, chronic diseases, body mass index and frequency of tooth brushing. Model 2 adjusted for education, socioeconomic status, age, chronic diseases and body mass index. Model 3 adjusted for education, socio-economic status and age.

**Results:**

The mean DMFT of 8294 women was 20.99 ± 8.95. In model 1, there was no significant relationship between DMFT and frequency of pregnancy. However, model 2 and 3 showed that in women who had four or more pregnancies, the DMFT rate was significantly higher than those who did not have a history of pregnancy (*P* = 0.02, *P* = 0.04). Age at the onset of menopause, age at the onset of menstruation and age of first pregnancy had no significant relationship with DMFT in the models. Brushing less than once a day and increasing age at interview had significant relationship with DMFT in the models (*P* < 0.001).

**Conclusion:**

Despite hormonal changes through the life, the history of reproductive showed no significant relationship with women’s DMFT. Oral health education for women is an important step in promoting oral health and it is necessary to pay special attention to preventive programs in oral health policy for women specially with increasing the age.

## Background

Women experience different hormonal changes through the life including menstruation, pregnancy and menopause. Oral health in women may be affected by these hormonal changes. Special attention to women’s oral health is essential to improving the quality of life [1]. Pregnancy can cause inflammatory changes in the physiological system [1]. Also, poor oral hygiene, and the increasing of dental caries and periodontal disease were reported in pregnant women [1]. Nutritional habits in the pregnant women often changes to include more sweet snacks, without change in daily oral hygiene habits [2]. On the other hand, during pregnancy, due to hormonal changes, women are reluctant to follow daily oral hygiene such as brushing and flossing, and using toothpaste, so maintaining good oral hygiene is a challenge for pregnant women [2]. Decayed teeth are prevalent among Iranian pregnant women [3]. The results of Persian cohort study in Mazandaran (Tabari cohort), showed that the rate of DMFT index increases with increasing the number of pregnancies [4]. In American women, there was no significant relationship between the number of children and the sum of filled and decayed surfaces. However, women with more children showed higher untreated caries [5]. In one study, the women who had their first childbirth at a young age, had significantly higher dental caries than other women [6]. However, in another study, age at the first pregnancy had no significant relationship with DMFT index [4]. Menopause indicates the end of a women’s reproductive stage in life and the start of a period of decreased estrogen [7]. Decreased estrogen production in postmenopausal period leads to many physiological changes, including increased susceptibility to dental caries [8] and higher DMFT scores [9]. Some studies showed that the gingival inflammation increased during menstrual cycle [10].

There are controversial results on the relationship between DMFT index and pregnancy. Various studies showed different risk factors for dental caries during pregnancy [11–13]. Therefore, this issue needs more comprehensive research. To our knowledge, there are no evidence about the relationship between dental caries and age at the onset of menstruation and menopause. Investigation of the effect of reproductive history in one population over time has not yet been extensively studied, and insufficient evidence is available in this field, especially in a comprehensive study. Consequently, we aimed to determine the relationship between reproductive history and DMFT index, based on the data obtained from Azar cohort study [14].

## Methods

### Study setting

This descriptive analytical cross-sectional study used the data obtained from the enrollment phase of the Azar cohort study which was a part of the Prospective Epidemiological Research Studies (PERSIAN cohort) for Iranian adults [15]. In 2014, Azar cohort sample was selected from permanent residence in Shabestar city in East Azerbaijan province, Iran About 15,000 participants with the age range of 35 to 70 years were included, which about 55% of whom were women. Azar cohort, a large population-based cohort study, has conducted for adults to assess risk factors for non-communicable diseases [14]. The assessors performed the interviews using predefined and validated research protocols and questionnaires of Persian cohort [14]. All eligible adults of the city, aged 35 to 70 years, were invited to participate in Azar cohort. Anyone who accepted the aim and steps of the study, filled a written informed consent and was free to leave the study at any time. The objectives of Azar cohort, sampling method, variables of the Azar cohort, and data collection tools and methods were explained in previous published papers [14, 15]. In our cross-sectional study, the inclusion criteria were the data of all women participating in the Azar cohort with a history of reproduction and oral health, while the exclusion criteria were the data of women lacking a history of reproduction and oral health. Consequently, the data of all 8294 women from the enrollment phase of the Azar cohort were entered into the analysis using the census method. This cross-sectional study was approved by the Ethics Committee of the Tabriz University of Medical Sciences, Tabriz, Iran (IR.TBZMED.REC.1400.1017).

### Data gathering

Data collection in the Azar cohort was performed through 4 forms. We extracted the necessary data for the present study from three of those forms. First, the “General information” form that covered sociodemographic characteristics. Socioeconomic (SES) status and educational level as the qualitative variable were extracted. In terms of SES, the participants were categorized into 5 groups (very poor, poor, medium, good, very good). Socioeconomic status was determined on the basis of job title, car ownership, number of trips made (per year), type of travel, having a personal computer, home ownership and having multiple jobs. In terms of educational level, the participants were categorized into 4 groups (illiterate, primary, diploma, university). Age at interview and BMI as the continuous data were reported by mean and standard deviation.

The second was medical history form which the participants were asked about history of different non-communicable diseases in their life. Self-report history of diabetes mellitus, hypertension, lung disease, depression, and cardiovascular disease were extracted from this form. The third was the “Oral health” and “reproductive history” form. “Oral health” information had two parts. First, the participant answered the questions about oral hygiene habits. We extracted the answer of this question for our study: “How often do you brush your teeth?” and divided the answers into two groups: brushing at least once a day and brushing less than once a day. Oral examination was done according to the World Health Organization (WHO) Oral Health Surveys Basic Methods [16] by a single examiner (a general practitioner who was trained by a skilled dentist), and the DMFT index was recorded properly using headlight, intraoral mirror, an explorer probe, and a piece of sterile gauze to clean teeth surfaces [17]. In this form, the participants were also asked about age at the onset of menstruation, age of onset of menopause, age of first pregnancy, frequency of pregnancy. The number of pregnancies was evaluated as a categorical variable in five groups with zero, one, two, three, four and more pregnancies. The age at the onset of menstruation was evaluated as a categorical variable in three groups: early (< 12), normal (12–14 years old), late (14<) [18]. The normal group was considered as the reference group. The age at the onset of menopause was evaluated as a categorical variable in three groups: Premature (< 40), early (40–45), normal (> 45). The normal group was considered as the reference group and the premature and early groups were compared with the normal group. The age of the first pregnancy was categorical variable in four age groups: 10–19, 20–29, 30–39, ≥40 which the reference group was 20–29. In our study, the variables related to the reproductive history were the exposure variables, and DMFT was the outcome variable. The other variables were considered as the possible confounding variables.

### Statistical analysis

For analysis, negative binomial analysis with loglink was applied. To adjust the effect of possible confounding variables, such as age at interview, educational level, socioeconomic status, frequency of tooth brushing, chronic diseases and body mass index along with the main variable were analyzed in several different regression models. Unadjusted model analyzed without considering the confounding variable. Model 1 adjusted for educational level, socio-economic status, age at interview, chronic diseases (diabetes mellitus, hypertension, cardiovascular, depression and lung diseases), body mass index, and frequency of tooth brushing. Model 2 adjusted for educational level, socioeconomic status, age at interview, chronic diseases (diabetes mellitus, hypertension, cardiovascular, depression and lung diseases), and body mass index. Model 3 adjusted for educational level, socio-economic status and age at interview. Statistical tests were carried out using SPSS 20.0 software (IBM Company, Chicago, IL, USA). Statistical significance was set at *p* < 0.05.

## Results

In the present study, the data of all 8294 women from Azar cohort study were analyzed. The data of menstrual age was available for 8286 women (8 missing data). The mean age of the participants was 49.24 ± 9.27. The percentage of participants in the age groups of 35–39, 40–49, 50–59, and 60–70 years, were respectively as follow: 18, 37, 29, and 16%. The mean DMFT index was 20.99 ± 8.95 which the missing teeth was the biggest part (DT = 1.71 ± 2.93, MT = 16.67 ± 11.44, FT = 2.61 ± 3.91). The mean BMI was 29.95 ± 5.10. Half of the women had four or more pregnancies. About 95% of first pregnancies are reported under 29 years of age. More than 40% of women had primary educational level. For 36% of women, socioeconomic status is reported to be good and very good. Blood pressure and depression were prevalent among women. Descriptive results are shown in Table 1.

**Table 1:** Distribution of participants based on reproductive history, socio-economic status, chronic diseases, tooth brushing

Variable		N	%
Frequency of pregnancies	0	452	5.5
	1	327	3.9
	2	1592	19.2
	3	1770	21.3
	4 ≤	4153	50.1
Age of the first pregnancy (years)	10–19	4028	51.4
	20–29	3409	43.5
	30–39	28	4.8
	≤40	377	0.4
Educational level	Illiterate	1857	23.7
	Primary	3189	40.7
	Diploma	2345	29.9
	University	445	5.7
Socioeconomic status	Very poor	2064	26.3
	Poor	1377	17.6
	Medium	1569	20
	Good	1663	21.2
	Very good	1163	14.8
Self-reported chronic and systemic diseases	Diabetes mellitus	1022	13
	Hypertension	1971	25.2
	Lung diseases	329	4.2
	Depression	1939	24.8
	Cardiovascular diseases	330	4.2
Frequency of tooth brushing	At least once a day	4386	52.9
	Less than once a day	3908	47.1
The onset of menstrual (years)	Early (< 12 year)	648	7.8
	Normal (12–14 years)	4911	59.3
	Late (> 14)	2727	32.9
The onset of menopause (years)	Premature (< 40)	215	6.2
	Early (40–45)	964	27.6
	Normal (> 45)	2314	66.2

The relationship of the frequency of pregnancies, the age at the onset of menstruation, the age at the onset of menopause, and the age of the first pregnancy with DMFT were assessed. The results from the unadjusted model and three adjusted models using Negative binomial regression analysis with log link were reported by modulating the effect of possible confounding variables, which are shown in Table 2.

**Table 2:** Relationship between reproductive history and DMFT index based on Negative binomial regression analysis

Variable		Unadjusted^1^			Adjusted								
					Model 1^2^			Model 2^3^			Model 3^4^		
		IRR^5^	95% CI	*P*-value	IRR	95% CI	*P*-value	IRR	95% CI	*P*-value	IRR	95% CI	*P*-value
Frequency of pregnancy	4 ≤	1.34	1.21–1.48	< 0.001	1.07	0.96–1.18	0.21	1.13	1.02–1.26	0.02	1.12	1.01–1.24	0.04
	3	1.04	0.93–1.15	0.49	1.07	0.96–1.19	0.2	1.11	0.99–1.23	0.07	1.09	0.98–1.22	0.10
	2	0.94	0.84–1.05	0.27	1.05	0.94–1.17	0.34	1.08	0.97–1.21	0.16	1.07	0.96–1.19	0.21
	1	0.9	0.78–1.04	0.18	1	0.87–1.16	0.96	1.01	0.87–1.16	0.95	1	1.86–1.16	0.99
	0	Reference											
Age at the onset of menstrual (years)	Early (12>)	1.06	0.98–1.16	0.15	0.99	0.91–1.08	0.91	1.01	0.93–1.09	0.84	1.01	0.93–1.09	0.86
	Late (14<)	1.12	1.07–1.18	< 0.001	1.01	0.96–1.06	0.77	1.02	0.97–1.07	0.34	1.03	0.98–1.08	0.25
	Normal (12–14)	Reference											
Age at the onset of menopause (years)	Precursor (< 40)	0.95	0.83–1.1	0.51	0.98	0.85–1.14	0.83	0.98	0.85–1.13	0.81	0.98	0.85–1.13	0.81
	Early (40–45)	1	0.93–1.08	0.99	1	0.93–1.08	0.95	1.01	0.94–1.09	0.77	1.01	0.94–1.09	0.75
	Normal (> 45)	Reference											
Age at first pregnancy (years)	40–49	0.95	0.65–1.39	0.78	0.89	0.61–32/1	0.58	0.9	0.61–1.32	0.59	0.9	0.62–1.32	0.6
	30–39	0.93	0.83–1.03	0.17	0.98	0.88–1.09	0.75	0.96	0.86–1.08	0.51	0.97	0.86–1.08	0.53
	10–19	1.1	1.05–1.16	< 0.001	1.01	0.96–1.06	0.71	1.01	0.97–1.07	0.55	1.01	0.96–1.06	0.76
	20–29	Reference											

Considering the relationship between the frequency of pregnancies and DMFT, the results of unadjusted model (*P* < 0.001), model 2 (*P* = 0.02) and model 3 (*P* = 0.04) showed that in women who had four or more pregnancies, the DMFT rate was significantly higher than those who did not have a history of pregnancy. However, in model 1, there was no significant relationship between DMFT and frequency of pregnancy. In this model, increasing age at interview (*P* < 0.001, IRR = 1.01) and, tooth brushing less than once a day (*P* < 0.001, IRR = 1.68) increased the DMFT scores.

In the unadjusted model, DMFT was significantly higher in women with late menstrual age than women with normal menstrual age (*P* < 0.001). In the adjusted models, there was no significant relationship between DMFT and age at the onset of menstruation. However, increasing age at interview (*P* < 0.001, IRR = 1.01), brushing less than once a day (*P* < 0.001, IRR = 1.69) and the low level of education (*P* < 0.001, IRR = 1.29) increased the DMFT scores in the models.

In all the models, the age at the onset of menopause had no significant relationship with DMFT. However, in these models, the results showed that tooth brushing less than once a day (*P* < 0.001, IRR = 1.69) and increasing age at interview (*P* < 0.001, IRR = 1.02) increased the DMFT scores in the models.

The unadjusted results showed that in women whose first pregnancy was between 10 and 19 years old, DMFT was significantly higher than others (*P* < 0.001). However, there was no significant relationship between DMFT and age of first pregnancy in the other models. Increasing age at interview (*P* < 0.001, IRR = 1.01) and, brushing less than once a day (*P* < 0.001, IRR = 1.67) increased the DMFT scores in the models.

## Discussion

This study was the comprehensive assessment of the relationship between reproductive history and DMFT based on the data obtained from Azar cohort study. In this study, there was no significant relationship between DMFT index and reproductive history (including age at the onset of menstruation, age of onset of menopause, age of first pregnancy, frequency of pregnancy) in all the adjusted models; except in model 2 and model 3, that women who had four or more pregnancies, had significantly higher DMFT compared to those who did not have a history of pregnancy.

In the present study, less than half of women brushed their teeth daily, while Sistani et al. (2017) reported 81.3% of adults in Tehran brushed their teeth daily [19]. According to Marla et al. (2018) study, about 56% of women did not seek dental treatment during pregnancy and only 35% had a dental visit during the first year following child-birth [20]. The behavioral changes during pregnancy that can cause dental caries and periodontal disease are common. Martins et al. (2014) showed that pregnant women brushed their teeth less frequently compared to pre-pregnancy. They also consumed more snacks and meals per day during pregnancy compared to pre-pregnant time [21]. The results of a study by Kamate et al. (2017) Showed that the third trimester of pregnancy and the postpartum period are periods in which pregnant women are at greater risk for dental caries [22]. Deghatipour et al. (2019) reported the high prevalence of gingival diseases and dental caries with an average of seven decayed teeth in Iranian pregnant women [3].

In the present study, the findings of model 2 and model 3 showed that women with ≥4 pregnancies had higher DMFT than other women, which is in line with the findings of the study of Gorji et al. (2021) in Mazandaran which showed that the average DMFT index in women increases with increasing number of pregnancies [4]. However, they did not assess the frequency of tooth brushing as a confounding factor in the analysis. Also, it is in agreement with the findings of Allameh et al. (2014) which demonstrated a significant association between the number of previous pregnancies with DMFT [13]. Russell et al. (2010) showed that in American women there was no significant relationship between the number of children and the sum of filled and decayed surfaces. However, women with more children showed higher untreated caries [8]. In our study, the model which we had entered frequency of tooth brushing along with other confounding factors in it (model 1) showed no significant relationship between the number of pregnancies and DMFT index. This can be indicated that if the mothers maintain good oral hygiene, the number of pregnancies will not have negative effect on DMFT index. Also in Geisinger et al. (2013) study, a statistically significant proportion of pregnant women had an excellent oral health status after receiving oral hygiene instructions [23]. Therefore, oral hygiene instructions for women has a very important role in promoting their oral health. Oral health related quality of life in pregnant women seems to be positively improved by the preventive oral programs during pregnancy [24]. Therefore, public policies and oral health programs are essential for improving oral health behaviors of pregnant women.

Age at first pregnancy may be associated with metabolic syndrome among middle-aged and older women [25]. In pregnant adolescents, caries experience and symptoms of periodontal disease were common [26]. The relationship between DMFT and age at first pregnancy is controversial. In the study of Gorji et al. (2021) no significant relationship was reported between the age at first pregnancy and DMFT index [4]. This was consistent with the findings of our study. Lee et al. (2020) reported increased chewing inconvenience and dental caries in women whose first delivery occurred at a young age [6] which is inconsistent with our results.

Women’s average menopause age differs in different parts of the world [27]. In Chinese women, dietary and reproductive characteristics, socio-demographic, lifestyle factors are related to the age at menopause [28]. Muka et al. (2016) showed a higher risk of coronary heart disease (CHD), cardiovascular diseases (CVD) and overall mortality in women who experience premature or early-onset menopause [29]. Previous studies have shown that physiological changes in postmenopausal women cause dry mouth which can lead to dental caries in postmenopausal women [9, 30]. Rafiei et al. (2022) found no correlation between hormonal fluctuations and periodontal status in postmenopausal women in Rafsanjan Cohort Study [31]. To our knowledge our study was the first one to assess the relationship between dental caries and age at the onset of menopause. We found no association between DMFT and age at the onset of menopause. However, in the models, increasing age and frequency of tooth brushing were statistically significant factors for higher DMFT index in postmenopausal women. Thus, oral hygiene instructions are important in the whole life specially in postmenopausal period.

Problems related to menstruation among adolescents can affect their social life. Dysmenorrhea is the most common menstrual problem which affects school performance and attendance [32]. Sargolzaee et al. (2013) showed that during menstrual cycle, gingival inflammation is increased and oral health should be maintained [10]. In the present study, there was no significant relationship between DMFT and age at the onset of menstruation. However, increasing age, tooth brushing, and the level of education had a strong relationship with DMFT in the models.

In total, in the present study, there was no significant relationship between reproductive history and DMFT index. However, the significant relationship of age at interview, and frequency of tooth brushing with DMFT in the models, emphasizes the role of oral hygiene in the whole life.

### Strengths

It was the first comprehensive evaluation of reproductive history in relation to DMFT index in a large sample size. The results can provide proper generalizability for Iranian people specially in northwest of Iran due to cultural and genetical similarities. A major strength of the present research is the adjustment of different potential confounders such as educational level, socio-economic status, age at interview, chronic diseases (diabetes mellitus, hypertension, cardiovascular, depression and lung diseases), body mass index, and frequency of tooth brushing.

### Limitations

Due to limitations of cross-sectional study, the data regarding the history of tooth brushing, flossing, using mouthwash, dental care and diet habits since childhood and during and before pregnancy, were not available. However, it will not affect the final relationship and conclusion of the study because it equally affects all participants of the study. Also, recall bias may occur in all cross-sectional studies, especially in large population-base studies.

## Conclusion

Considering our results, it seems that the history of reproductive has no significant relationship with women’s DMFT index. The role of oral hygiene throughout life is emphasized as an important factor in promoting oral health. Oral health education for women in the community is an important step in promoting their oral health and it is necessary to pay special attention to preventive programs in oral health policy for women, especially with increasing the age. In the further studies, assessing the gingival index or bleeding on probing along with DMFT are suggested to investigate the relationship between oral health and the history of reproductive. Also, it would be recommended to conduct a study among pre-menopause women to assess the effect of age at menstruation, age at the first pregnancy and the frequency of pregnancies on DMFT index.

## Data Availability

The datasets used and/or analyzed during the current study are available from the corresponding author on reasonable request.

## Abbreviations

DMFT: Decayed, Missing, and Filled Teeth in the permanent dentition
DT: number of Decayed Teeth in the permanent dentition
MT: number of Missing Teeth due to caries in the permanent dentition
FT: number of Filled Teeth in the permanent dentition
PERSIAN: Prospective Epidemiological Research Studies in Iran
BMI: Body Mass Index
IRR: Incidence Rate Ratio
